# Violence against civilians and access to health care in North Kivu, Democratic Republic of Congo: three cross-sectional surveys

**DOI:** 10.1186/1752-1505-4-17

**Published:** 2010-11-08

**Authors:** Kathryn P Alberti, Emmanuel Grellety, Ya-Ching Lin, Jonathan Polonsky, Katrien Coppens, Luis Encinas, Marie-Noëlle Rodrigue, Biagio Pedalino, Vital Mondonge

**Affiliations:** 1 Epicentre, 8 rue Saint Sabin, 75011 Paris, France; 2 Médecins Sans Frontières Holland, Lubumbashi, Democratic Republic of Congo; 3 Médecins Sans Frontières Holland, Plantage Middenlaan 14, 1001 EA Amsterdam, The Netherlands; 4 Médecins Sans Frontières Belgium, rue Dupré 94, 1090 Brussels, Belgium; 5 Médecins Sans Frontières France, 8 rue Saint Sabin, 75011 Paris, France; 6 Ministry of Health, Democratic Republic of Congo, Gombe, Kinshasa, Democratic Republic of Congo

## Abstract

**Background:**

The province of North Kivu in the Democratic Republic of Congo has been afflicted by conflict for over a decade. After months of relative calm, offences restarted in September 2008. We did an epidemiological study to document the impact of violence on the civilian population and orient pre-existing humanitarian aid.

**Methods:**

In May 2009, we conducted three cross-sectional surveys among 200 000 resident and displaced people in North Kivu (Kabizo, Masisi, Kitchanga). The recall period covered an eight month period from the beginning of the most recent offensives to the survey date. Heads of households provided information on displacement, death, violence, theft, and access to fields and health care.

**Results:**

Crude mortality rates (per 10 000 per day) were below emergency thresholds: Kabizo 0.2 (95% CI: 0.1-0.4), Masisi 0.5 (0.4-0.6), Kitchanga 0.7 (0.6-0.9). Violence was the reported cause in 39.7% (27/68) and 35.8% (33/92) of deaths in Masisi and Kitchanga, respectively. In Masisi 99.1% (897/905) and Kitchanga 50.4% (509/1020) of households reported at least one member subjected to violence. Displacement was reported by 39.0% of households (419/1075) in Kitchanga and 99.8% (903/905) in Masisi. Theft affected 87.7% (451/514) of households in Masisi and 57.4% (585/1019) in Kitchanga. Access to health care was good: 93.5% (359/384) of the sick in Kabizo, 81.7% (515/630) in Masisi, and 89.8% (651/725) in Kitchanga received care, of whom 83.0% (298/359), 87.5% (451/515), and 88.9% (579/651), respectively, did not pay.

**Conclusions:**

Our results show the impact of the ongoing war on these civilian populations: one third of deaths were violent in two sites, individuals are frequently subjected to violence, and displacements and theft are common. While humanitarian aid may have had a positive impact on disease mortality and access to care, the population remains exposed to extremely high levels of violence.

## Background

A 5-year war that ravaged the Democratic Republic of Congo (DRC) officially ended with the endorsement of peace agreements and withdrawal of troops in 2003. The war had a devastating impact; millions of civilians died, many due to lack of access to health care[1-5]. Although a national peace process has been held and elections were conducted in 2006, the eastern regions of the country have yet to see the end of hostilities and in 2009, for the 11^th ^consecutive year, DRC was featured on a list of the Top Ten Humanitarian Crises[6].

In the province of North Kivu, in eastern DRC, conflict has never ceased. Intermittent fighting continues between the national army and multiple, shifting rebel groups. Frontlines move, leading to continual instability and population displacement. In August 2008, after months of relative calm, offensives restarted, resulting in mass population displacement.

The non-governmental organisation (NGO) Médecins Sans Frontières (MSF) has run health-care programmes in this region for many years. Services provided in collaboration with the Ministry of Health vary between sites, and can include primary and secondary health care, emergency surgery, therapeutic treatment for malnourished children, response to epidemics (eg, cholera), mental health care, and care for individuals subjected to sexual violence. These services are provided for both internally displaced persons (IDPs), mainly living in camps, and for resident populations. Additional health services and support, such as the provision of water and latrines, and the distribution of essential non-food items such as jerry cans and plastic sheeting, is provided by a range of actors (Solidarité, Merlin, MSF, UNICEF, Save the Children).

In May 2009, to understand the impact of the renewed conflict on selected civilian populations, and to assess their access to health care in order to orient pre-existing humanitarian aid, surveys were carried out in the catchment areas of three MSF programmes in North Kivu. They covered the period from the last major offences in September 2008 to the time of the surveys in May 2009.

## Methods

Three cross-sectional surveys were undertaken, one in each of three geographic areas supported by MSF. Both IDP camps and local villages were included.

### Setting

The three sites--Kabizo, Masisi, and Kitchanga--all lie to the northwest of Goma, the capital of North Kivu. The sites are geographically close (less than 100 km between sites), but all have distinct histories. All sites were affected by the renewed fighting as of September 2008. Kabizo town was directly attacked, with the population fleeing to a nearby town and pre-existing IDP camp. In Masisi, the town itself was not the scene of direct fighting, but clashes in the surrounding villages lead to continual displacement, returns, and the creation of new IDP camps. In Kitchanga, the population of a small town has been outnumbered by IDPs in two camps. Additionally, as the ongoing conflict continues in the region, more IDPs are settling further to the north of Kitchanga. In all sites, the MSF health programme, run in collaboration with the Ministry of Health, had been in place for at least 1 year prior to the most recent clashes.

### Sample size calculation

Sample size was calculated to estimate a crude mortality rate of 0.7 deaths/10 000 people/day over a period of 8 months, with a precision of 0.3. The point estimate was based on the results of a rapid assessment carried out in another area of North Kivu in February 2009. Assuming an average household size of 5, a minimum of 249 households were required for each survey using systematic sampling. Assuming the same household size and a design effect of 3, 747 households were required for each cluster sampling survey.

### Survey procedures

Surveyors were given 2 days of theoretical and 1 day of practical training in the field prior to starting the survey. The three epidemiologists responsible for the surveys all participated in the first training to ensure consistency between sites.

In Kabizo town and camp, where the households were arranged in a semi-regular pattern, systematic sampling was used, with the sampling interval calculated based on the most recent population estimates available. Town population data were obtained from local authorities (2008 census). NGOs provided 2009 population data for IDP camps. The sampling interval (x) was calculated by dividing the total estimated number of households by the required number of households based on the sample size calculation. The first household in each camp or town section was selected by choosing a random number between 1 and x. The sampling interval was then added to this random number to select the next household with the procedure repeated until the end of the section. Local outreach workers were used as guides to ensure that no houses were missed and that section boundaries were respected. One village was excluded from the survey for security reasons.

In Masisi and Kitchanga, two-stage cluster sampling was used following the standard World Health Organisation (WHO) Expanded Programme on Immunization cluster sampling proximity method[7]. Probability of selection was proportional to population size based on the smallest possible geographic area (village or camp section) and the most recent census data (Masisi January 2007 census with 3.0% growth adjustment, Kitchanga 2009 NGO estimates). In Masisi, any area with active fighting or where distance would have required the surveyors to remain in the village overnight was excluded.

In Masisi, the initial household in each cluster was selected using the EPI 2 method[8]. Because of the long distances to travel to reach each cluster site in Masisi, we estimated that 20 households could be interviewed per team per day and the cluster size was defined accordingly. In Kitchanga, the initial household in each cluster was the household closest to a randomly selected global positioning system (GPS) point from within the mapped cluster area. Travel to distant sites was facilitated by the set-up of a sub-base where teams could spend the night, thus reducing daily travel times. We estimated that 25 households could be interviewed per team per day, and the cluster size was defined accordingly.

A household was defined as a group of people under the responsibility of one person (head of household) and who share meals.

If a house was found empty, neighbours were asked to help establish whether the house was abandoned or inhabited, and if inhabited, to find absentees. Survey team members returned at least once to each house to try to find absentees. In the event of absence, refusal, or if no adult (older than 15 years) was present, the house was skipped and replaced with the next nearest house.

The same basic survey questionnaire was used in all three sites, with minor changes made to the recall period to adapt to local events. The questionnaire was written in French, translated into Swahili, and back-translated to French for verification. All surveyors spoke French, Swahili, and/or other local languages. The purpose of the survey was explained to heads of households and written consent (thumbprint or signature) obtained before beginning the interview. Data were collected on household members who had been present at the beginning of the recall period, including those who had since left, resident household members, and household members who had died during the recall period (including babies who were born and died during the period). Age and sex of all individuals, either currently alive or deceased during the recall period, were collected as well as dates of births, deaths, arrivals in, or departures from the household. Information on the reported cause of death was also collected. No detailed verbal autopsy or other verification was done for reported cause of death.

Data on violence against individuals, both the number and type of episode, were collected. If a household member had experienced an episode of violence, they were asked to give the nature of the violence (beating, rape, injury by firearm or other weapon, detention, abduction, forced labour, or extortion). One response could be given for each episode. Individuals were also asked when the episode occurred and to describe the perpetrator from a list of categories (combatant, "incivique" [a local term used for a deserter], civilian, unknown, no response). Each episode during the recall period was described separately.

In addition to individual data, data were collected at the household level on current status (displaced, returnee, resident), number of displacements during the recall period, reason for displacement, distance of displacement (measured in number of days walked), theft of essential household items (jerry can, cooking set, blanket, livestock, or food-stocks), and access to agricultural fields.

To estimate access to health care, information was collected on the last household member to be ill, if the illness occurred during the 2 weeks before the survey. Information on care seeking and payment for services was collected for these individuals.

Questionnaires were checked for completeness and accuracy at the end of each day. Data were entered in EpiData 3.0 (EpiData Association, Odense, Denmark) with 10% general data checks and all major events verified. STATA 10 (StataCorp, College Station, TX), EpiInfo 6.04 d (Centers for Disease Control and Prevention, Atlanta, USA and WHO, Geneva, Switzerland) and Emergency Nutrition Assessment (ENA) for SMART http://www.nutrisurvey.de/ena/ena.html were used for data analysis. For the Masisi and Kitchanga analyses, software or commands that estimate design effect and incorporate them in confidence interval calculations were used.

### Ethics statement

Ethical approval was obtained from the Ethical Committee of the School of Public Health, Kinshasa, Democratic Republic of Congo.

## Results

The results of these surveys are representative of an estimated population of 200 000 inhabitants. The surveys were conducted between May 4 and May 23 2009. In Masisi, one cluster could not be completed because fighting broke out in the village where the survey was being conducted and the survey team was forced to evacuate. The cluster was not replaced. In Kabizo, eight households (1.5%) refused to participate in the survey and 48 (8.0%) were absent; in Masisi nine (1.0%) refused and 29 (3.0%) were absent; and in Kitchanga 18 (1.8%) refused and 55 (5.1%) were absent.

The main characteristics of the surveyed population are shown in Table 1. The overall male/female ratio in each survey site was 0.9. The average household size in Kabizo and Kitchanga was 5.3, and in Masisi was 6.6. The proportion of the population under the age of 5 years ranged from 17.5% to 19.7% in the three sites.

**Table 1:** Description of survey populations

			< 5 years			Total population		
	n clusters	n families	Male	Female	Total	Male	Female	Total
Kabizo	n/a	542	276	258	534	1382	1476	2858
Masisi	48	905	507	536	1043	2839	3123	5962
Kitchanga	42	1075	514	552	1066	2558	2858	5416

At each site, the majority of households reported either being displaced at the time of the survey (including displacement prior to the 8-month recall period) or being displaced during the recall period and having since returned home (Table 2). Households also reported being displaced more than once during the recall period. The most commonly reported reason for displacement during the recall period was direct attack on their village: Kabizo 81.3% (216/346, 95% CI: 76.7 - 86.2); Masisi 85.6% (951/1117, 95% CI: 72.8 - 91.2); and Kitchanga 60.7% (366/608, 95% CI: 60.3 - 70.1). The average distance of displacement, in days travelled, for those displaced during the recall period (excluding those who have since returned home) was: Kabizo 0.6 days (95% CI: 0.6 - 0.7), Masisi 1.6 days (95% CI: 1.3 - 1.9), and Kitchanga 1.3 days (95% CI: 1.1 - 1.5).

**Table 2:** Families reporting displacement during September 2008 - May 2009 and those self-reporting status of displaced May 2009

	Displaced during recall period			Current displaced		
	n	%	95% CI	n	%	95% CI
Kabizo	274	50.6	46.6 - 55.1	258	47.6	43.5 - 52.1
Masisi	903	99.8	99.4 - 100.0	376	41.5	32.6 - 51.0
Kitchanga	419	41.1	31.7 - 50.6	898	88.1	79.3 - 93.5

The crude and under-5 mortality rates (CMR, U5MR) are shown in Table 3.

**Table 3:** Crude and under-5 mortality rates (CMR, U5MR) per 10 000 per day, September 2008 - May 2009

	n	CMR	95% CI	Design Effect	n	U5MR	95% CI	Design Effect
Kabizo	15	0.2	0.1 - 0.4	n/a	8	0.7	0.4 - 1.5	n/a
Masisi	68	0.5	0.4 - 0.6	1.8	23	1.0	0.7 - 1.5	1.0
Kitchanga	92	0.7	0.6 - 0.9	2.3	38	1.6	1.2 - 2.2	1.3

In Kabizo and Masisi, the CMR and U5MR did not exceed established emergency thresholds (CMR > 1 death/10 000 people/day, U5MR > 2 deaths/10 000 children under 5/day)[9]. In Kitchanga, the upper 95% CI of the U5MR exceeded the emergency threshold for this age group. In both Masisi and Kitchanga, the most commonly reported cause of death was violence (Table 4). Although less frequent than in adults, violent deaths were also reported in the under-5 populations in Masisi and Kitchanga (Table 4). The deaths reported in our surveys suggest that between 650 and 1030 violent deaths occurred between September 2008 and May 2009 in these three populations, with most occurring in Masisi and Kitchanga.

**Table 4:** Reported number and causes of death by age group, September 2008 - May 2009

	< 5 years			≥ 5 years			Total		
	Total	Violent	Non-violent	Total	Violent	Non-violent	Total	Violent	Non-violent
	**N**	**n (%)**	**n (%)**	**n**	**n (%)**	**n (%)**	**n**	**n (%)**	**n (%)**

Kabizo	8	0(0#183;0)	8(100#183;0)	7	2(28#183;6)	5(71#183;4)	15	2(13#183;3)	13(86#183;7)
Masisi	23	8(34#183;8)	15(65#183;2)	45	19(42#183;2)	26(57#183;8)	68	27(39#183;7)	41(60#183;3)
Kitchanga	38	4(10#183;5)	34(89#183;5)	54	29(53#183;7)	25(46#183;3)	92	33(35#183;9)	59(64#183;1)

In Kabizo, 92 episodes of violence against individuals were reported, including two women who reported being raped. In Masisi, 914 episodes were reported, including 103 rapes (11.3%, 95% CI: 8.2 - 16.1). In Kitchanga, 922 episodes were reported including 44 rapes (4.8%, 95% CI: 3.3 - 6.3). Forced labour was the most commonly reported type of violence against individuals in Masisi 41.9% (383/914, 95% CI: 35.3 - 48.7) and Kitchanga 51.5% (475/922, 95% CI: 44.1 - 58.9). Beatings were the most commonly reported act of individual violence in Kabizo: 66.3% (61/92, 95% CI: 54.7 - 76.2). At all sites, over 95% of perpetrators were reported to be a combatant (rebel or military) or an "incivique". In Kabizo, 11.6% (63/542, 95% CI: 8.8 - 15.3) of households reported at least one household member being subjected to violence during the recall period; in Masisi 99.1% (897/905, 95% CI: 98.2 - 100.0); and in Kitchanga 50.0% (509/1018, 95% CI: 46.8 - 54.1).

Theft or destruction of property was also common during the recall period. In Kabizo 87.7% (451/514, 95% CI: 84.4 - 91.2) of households reported theft or destruction of major items; in Masisi 96.0% (869/905, 95% CI: 92.2 - 98.0); and in Kitchanga 57.4% (585/1018, 95% CI: 48.8 - 65.6).

Access to fields was either not possible or limited for 55.4% (300/542, 95% CI: 51.5 - 59.2) of households in Kabizo, 41.3% (186/451, 95% CI: 32.9 - 50.3) in Masisi, and 79.7% (811/1018, 95% CI: 73.5 - 85.9) in Kitchanga.

At all sites, 70% of households reported having at least one member fall ill and require care outside the home in the 2 weeks prior to the survey. In Kabizo, 94.8% (364/384, 95% CI: 92.0 - 96.6) received care, of whom 84.4% (/359, 95% CI: 80.4 - 87.7) did not pay for their consultation or treatment. In Masisi, 81.7% (515/630, 95% CI: 75.9 - 87.5) of the sick received care, of whom 87.6% (451/515, 95% CI: 83.4 - 91.6) did not pay. In Kitchanga, 89.8% (651/725, 95% CI: 86.3 - 93.3) of the sick received care, of whom 88.9% (579/651, 95% CI: 86.5 - 92.8) did not pay.

## Discussion

Our results, obtained in insecure field conditions, show the ongoing impact of the conflict on civilian populations, in particular the violence inflicted upon them. Although mortality rates were under emergency thresholds in our surveys, the proportion of violent death was high, reaching over 30% in the global population in two sites and 58% and 71% in the population 5 years and over in the same sites. While the CMRs in our surveys were lower than that of other surveys undertaken in camps during acute crises (4.1/10 000/day in Ituri, DRC 2005), they are similar to results reported from eastern DRC as part of a nationwide survey carried out in 2006 - 2007 [4,10], The proportion of deaths reported as violent in two of our survey sites is similar to the 67% reported in the Ituri survey, but much higher than the 0.6% violent deaths, reported in the cumulative results of the eastern region in the 2006 - 2007 survey[4,10].

Our results may under-represent the true extent of deaths due to violence (and violence against individuals), since some villages in Kabizo and Masisi were excluded from the survey for security reasons. These sites, and that from which the team was evacuated, were likely to have been more strongly affected by violence than those in which the survey could be conducted. In addition, the MSF programmes cover a wider more inaccessible area than could be covered in the surveys. The programmes include areas only reachable by mobile teams supporting government health centres that cannot be accessed on a regular basis because of insecurity. These are populations that continue to be affected by conflict, are constantly moving, and likely have high levels of trauma and mortality, as yet unrecorded.

The geographic situation of Kabizo may be linked to the few reported deaths from violence. During the recall period of the survey the relatively large town of Kabizo was overrun and residents fled when the first skirmishes occurred, most escaping the violence; no further attacks occurred in the survey area. In Masisi and Kitchanga, numerous offensives were conducted on multiple villages, which might have led to less warning (as demonstrated by the unanticipated attack on the village in which one survey team was working), resulting in more fatalities.

We did not carry out verbal autopsies in the field. For that reason we present the reported causes of death as either violent or non violent, since we consider that respondents could reliably report this information.

Our results, unlike others obtained in eastern DRC, show mortality linked to disease well below emergency thresholds[2-4,10]. However, our survey sites were not representative of all of North Kivu and reflect the situation of only selected populations for whom humanitarian aid had been in place for at least a year. The medical care provided to these populations was comprehensive and free of charge, and our results suggest that access to care was good. Although not quantified in our survey, water and hygiene activities were also in place in all sites. Together, these activities could have contributed to the disease-related mortality being lower than that reported in other surveys, and demonstrate that high disease-related mortality rates are not inevitable.

The exposure of the population to violence is also revealed in the number of violent events perpetrated against individuals. Few are exempted, with nearly half the households included in our survey affected during the 8-month recall period.

The episodes of violence against individuals, particularly rape, are likely to have been under-represented in our survey results. Rape is still a taboo subject in many communities of DRC, and reporting can have severe negative consequences for the victim. In our survey nearly all perpetrators were identified as combatants, and there may be under-reporting of violence perpetrated within the community. Another limitation in the data on violence is that we only recorded one type of violence per violent episode. Although this limitation does not affect the number of episodes, it might under-represent what are considered less severe forms of violence--eg, an episode of violence might be recorded as rape when the person was subjected to both rape and beating.

Our results also reveal the prevalence of forced labour in two of the survey sites. Civilians, usually men, are used to carry material for combatants, frequently for long distances and usually under threat of violence if they do not comply. The frequency of forced labour in our survey areas is higher than reported previously[2].

The high proportion of households who had basic non-food items, livestock, or food-stocks stolen, and who had limited or no access to their fields exposes more of the precarious existence of these populations. While in these sites NGOs have generally replaced essential items, the same might not be true for other populations of North Kivu. The poor access to fields and loss of livestock also suggests that households may struggle to meet even basic needs in the short and medium terms. Although we did not collect specific information about why fields could not be accessed, it might be linked to the distance the population have been displaced. In insecure conditions, individuals are unlikely to stay overnight at their fields, which would be necessary if they are more than a few hours walk from their current residence.

Our results show the continual instability of the population, many of whom had fled at least once during the 8-month recall period, many more than once, and most as a result of direct attacks on their homes at the time of the displacement. This is in contrast to rumours within the international community that villagers had been fleeing pre-emptively, before combatants arrived. In general, the displaced did not flee far from their homes, most walking for less than 2 days before settling. An area of direct conflict may be located very close to an area where the population settles. The challenge faced by humanitarian organisations is to provide aid for the dispersed populations and to ensure the security of their teams.

The prevalence of violence as seen in our survey results is high and reflects what is reported by field teams. The MSF programmes see many survivors of sexual violence (in one site, an average of 68 per month from a population estimated at around 140 000; L Berryman, personal communication), although it is thought that the team manages to reach only a small proportion of survivors due to lack of access to information and issues of stigma and confidentiality. MSF mental-health programmes in the region treat people who have commonly experienced a combination of different traumatic events: having to flee and hide from enemies, houses being destroyed, family members, neighbours, and friends being killed or disappearing, witnessing someone being killed or raped, being raped, and properties, livestock, and fields being confiscated. Their main complaints are anxiety related, with sleeping disorders and intense psychological distress the most frequent symptoms (L Berryman, personal communication).

## Conclusions

The results of these surveys are both positive and negative. Positive, in that access to health care was good, and disease-related mortality was lower than shown in previous surveys. Negative, in that the direct consequences of violence were experienced by an extremely large proportion of the population. The civilian population suffers high levels of violence, is regularly displaced, and has property stolen. These results show that, although high disease-related mortality rates are not inevitable, the population of North Kivu continues to suffer the appalling effects of this devastating conflict, despite the peace agreements and elections.

## Competing interests

The authors declare that they have no competing interests.

## Authors' contributions

KPA participated in the design and interpretation of the study and wrote the paper; EG, YCL, and JP participated in the design of the study, analysis and interpretation of data, and revising the paper critically for substantial intellectual content; KC, LE, MNR, and BP participated in the conception and design of the study and revising the paper critically for substantial intellectual content; VM participated in the conception of the study and revised the paper critically for substantial intellectual content. All authors have read and approved the final manuscript.
